# The Role of CD4 and CD8 T Cells in Human Cutaneous Leishmaniasis

**DOI:** 10.3389/fpubh.2014.00165

**Published:** 2014-09-29

**Authors:** Claire da Silva Santos, Cláudia Ida Brodskyn

**Affiliations:** ^1^Centro de Pesquisas Gonçalo Moniz, Fundação Oswaldo Cruz (Fiocruz) Bahia, Salvador, Brazil; ^2^Faculdade de Medicina da Universidade Federal da Bahia, Salvador, Brazil; ^3^Instituto de Investigação em Imunologia – Instituto Nacional de Ciência e Tecnologia (iii-INCT), Salvador, Brazil

**Keywords:** adaptive immunity, human adaptive immunology, cutaneous leishmaniasis, CD4 and CD8 T cells, *Leishmania braziliensis*, immune response

## Abstract

Leishmaniasis, caused by infection with parasites of the *Leishmania* genus, affects millions of individuals worldwide. This disease displays distinct clinical manifestations ranging from self-healing skin lesions to severe tissue damage. The control of *Leishmania* infection is dependent on cellular immune mechanisms, and evidence has shown that CD4 and CD8 T lymphocytes play different roles in the outcome of leishmaniasis. Although the presence of CD4 T cells is important for controlling parasite growth, the results in the literature suggest that the inflammatory response elicited by these cells could contribute to the pathogenesis of lesions. However, recent studies on CD8 T lymphocytes show that these cells are mainly involved in tissue damage through cytotoxic mechanisms. In this review, we focus on the recent advances in the study of the human adaptive immunological response in the pathogenesis of tegumentary leishmaniasis.

Tegumentary leishmaniasis (TL) is transmitted by sand flies and is caused by different species of Old and New World *Leishmania*. The disease is characterized by a wide spectrum of clinical manifestations, including self-healing skin lesions, cutaneous leishmaniasis (CL), disseminated leishmaniasis (DL), mucosal leishmaniasis (ML), and diffuse cutaneous leishmaniasis (DCL) [reviewed in Ref. (1)]. Host–parasite interactions can lead to a series of events, culminating in the different forms of clinical manifestations. This review will address the role of adaptive immunological responses in the pathogenesis of TL.

## A Brief Summary of Innate Immune Responses in Infections Caused by *Leishmania*

In the early events after *Leishmania* infection, macrophages are activated, leading to NO synthesis (2–6), ROS production, and lysosomal enzyme activation (7, 8), which are responsible for killing the parasite. In contrast, the alternative activation of macrophages by TGF-β provides a favorable environment for *Leishmania* proliferation (9). Indeed, alternatively activated macrophages preferentially induce the arginase activity responsible for parasite replication (10). Recently, the importance of neutrophils in the first events after *Leishmania* infection was demonstrated. Using intravital microscopy, Peters et al. showed these cells are rapidly recruited to the infection site and are responsible for phagocytosis of the parasites (11). Furthermore, these cells undergo apoptosis, and the parasites released in this process are engulfed by macrophages, creating an anti-inflammatory environment that favors the establishment of infection. Our group also observed that *Leishmania amazonensis*-infected macrophages in the presence of resting apoptotic neutrophils produce TGF-β and PGE-2, leading to parasite replication. Conversely, the presence of necrotic neutrophils induces activation in infected macrophages, with a decrease in the number of parasites phagocytosed by macrophages (12). Human neutrophils were found to be activated by *L. amazonensis* via LTB-4 production, which promotes neutrophil degranulation and the killing of parasites (13). In addition, the release of neutrophil-derived extracellular DNA-containing antimicrobial peptides is an important neutrophil function that contributes to parasite killing (14, 15).

Dendritic cells (DCs) are critical for the initiation of an effective immune response against *Leishmania*. In the murine model of *Leishmania major*, DC infection leads to cell activation, which up-regulates costimulatory molecules, such as MHC II, CD80, and CD86, and the release of IL-12, antigen presentation and T cell priming (16, 17). Some reports have shown that infection with different species of *Leishmania* parasites does not lead to changes in the activations markers on DCs (18, 19). For instance, the infection of DCs by *Leishmania braziliensis* inhibits the up-regulation of activation markers and antigen presentation, whereas uninfected cells are able to up-regulate MHC class II and costimulatory molecules, inducing T cell activation (20). It was suggested that these DCs in *L. braziliensis* infection lead to T cell activation, with infected DCs contributing to parasite control through enhanced TNF-α production.

In human leishmaniasis, NK cells are found to accumulate rapidly at the inoculation site after *Leishmania* parasite invasion (21). These cells are an important source of interferon (IFN)-γ, which elicits microbicidal activity by macrophages. The protective role of NK cells in human leishmaniasis can be evidenced by the recruitment of NK cells into the lesions of DCL patients who respond to treatment (22, 23).

## Immune Responses Mediated by CD4 T Cells in Human Cutaneous Leishmaniasis

The cellular immune responses in leishmaniasis have been extensively studied in mouse models, mainly using *L. major* infection. Susceptible mice (BALB/c) develop progressive lesions, with a predominance of the Th2 response, leading to the production of anti-inflammatory cytokines, such as IL-4, IL-5, and IL-13. Resistant mice infected by *L. major* display small lesions with few parasites and a predominance of IFN-γ, TNF-α, and IL-2 cytokines, characteristic of a Th1 response. These latter cytokines activate leishmanicidal mechanisms in infected macrophages, with high ROS and NO production, leading to parasite killing (24).

In human beings, the immune response has an essential role in pathogenesis, and it is not possible to observe Th1 and Th2 polarization. The cytokine profiles produced by T cells are associated with the healing process (25–27) or with the development of disease (28–30), as well as protective mechanisms (31). The unresponsive pole of the disease observed in DCL is characterized by the high production of anti-inflammatory cytokines, such as IL-10, IL-4, and IL-2, but there is no IFN-γ production upon *in vitro* stimulus with *Leishmania* antigens (28). However, in ML, the responsive pole of the disease, the cells from patients display an exacerbated immune response (32) with a positive DTH (33–35). There is some evidence that the tissue destruction observed in LCL and ML is related to the immune response rather than to the parasites present in the lesions, the number of which is very low [reviewed in Ref. (1)].

Cells from ML patients stimulated *in vitro* with *Leishmania* antigens secrete higher concentrations of pro-inflammatory cytokines, such as TNF-α and IFN-γ, compared to cells from CL patients (32). These cytokines are also present in the lesions of ML patients (34), and TNF-α levels decrease following treatment. The immune response in CL patients reveals a mixture of cytokines, with the presence of anti- and pro-inflammatory cytokines in tissues. CD4 T cells are the major source of IFN-γ (36, 37), and this cytokine, as well as TNF-α, controls parasite multiplication during the early phases of *Leishmania* infection. Regardless, these cytokines also mediate the tissue damage (30). CD4 T cells at the site of skin lesions were found to be the key producers of IFN-γ upon restimulation *in vitro* and were capable of activating macrophages for the killing of intracellular parasites. Unlike CD8 T cells, CD4 T cells were reported not to be involved in the cytolysis of infected target cells. Therefore, no correlation between CD4 T cells and lesion size/immunopathology was found (38).

In CL patients, TNF-α is produced by different types of cells, including lymphocytes and macrophages (16). Recently, a positive correlation between ulcer size at the time of the first evaluation and TNF-α levels was observed, supporting the use of TNF inhibitors combined with standard therapy to improve recovery time in CL patients with severe lesions (39). The treatment of leishmaniasis patients with pentoxifylline associated with a pentavalent antimonial decreased the recovery time in CL and ML patients, even in those who were refractory to conventional treatment (40, 41).

IL-10 is also produced by CL patients and is responsible for down-regulating inflammatory responses, mainly those induced by IFN-γ (42–44). IL-10 is produced by a variety of cells, including macrophages, regulatory T (Treg) cells, Th1 cells, and CD8 T cells. The presence of Treg cells (natural and inducible) in the lesions from CL patients as well as IL-10 and TGF-β production has already been described. These cytokines are responsible for the control of the immune response in CL patients but also for the pathology of disease, deactivating the mechanisms of macrophage killing and leading to parasite persistence (45, 46). A down-regulation of IL-10 receptor was demonstrated in lesions from ML patients, which can partly explain the lack of IL-10 response and the absence of inflammatory process down-regulation (34).

More recently described cytokines are also observed in TL patients. IL-27, a cytokine with close structural and functional similarity to the IL-6/IL-12 family, is also expressed at high levels in the peripheral blood and tissues of ML and CL patients. Despite the induction of Th1 differentiation during the first steps of the immune response, IL-27 has been shown to promote an attenuation of inflammatory responses, improving IL-10 production by Th1 CD4 T cells (47–49). This function precludes inflammation and subsequent tissue damage in the late phase of the immune response. However, Oliveira et al. showed that the addition by IL-27 of PBMCs from CL and ML patients did not enhance IL-10 production by these cells, suggesting that IL-27 did not have an effect on regulating the strong inflammatory response observed in human CL patients (50).

To better understand the pathogenesis of TL, Carvalho’ group has studied SC patients, who display a positive DTH response against *Leishmania* antigens but do not exhibit any lesions. These patients are infected by *L. braziliensis*, but their immune responses are protective against the development of disease. Individuals with SC *L. braziliensis* infection present cellular immune responses that are less intense than those observed in CL or ML and produce significantly lower levels of IFN-γ and TNF-α than CL patients (51). More recently, Novoa et al. reported a stronger Th1 response in CL patients than in SC individuals, though the levels of IL-10 were higher in the latter patients than in the former patients (52). Although the mechanisms by which SC individuals control parasite growth are unknown, innate immune responses could play an important role in this control, with the participation of neutrophils, macrophages, and NK cells [reviewed in Ref. (53)].

More recently, the role of TH17 cells in the pathogenesis of TL has been discussed. Although the presence of these cells has been associated with the pathology of many autoimmune diseases, their role in leishmaniasis is not clear. The presence of Th17 cells and neutrophil recruitment are observed in the ML lesions of patients, in areas of necrosis and also with MMP-9 participation (54, 55). However, in CL patients, IL-17 is present in the supernatant of cells stimulated by *Leishmania* antigens; nonetheless, its role in the pathology of the disease is not well established (55). Therefore, different subsets of CD4 T cells not only produce cytokines responsible for the control of parasite proliferation but also contribute to the development of inflammatory responses. The lack of down-regulation of this inflammation could be responsible for the pathogenesis, as is observed in ML patients. However, as observed in SC patients, the balance of pro- and anti-inflammatory cytokines contributes to protection against the disease. In addition, there is recent evidence of the role played by CD8 T cells in the pathogenesis of the disease.

## CD8 T Cell Immune Response in *Leishmania* sp Infection

CD8 T cells provide immunity against a wide variety of pathogens, including viral, bacterial, and protozoal infections. These cells are generally considered to contribute to immunity and protection against *Leishmania*.

Muller et al. showed that the initial transitory depletion of CD4 T cells in mice susceptible to *L. major* infection causes them to be resistant to infection by the parasite via a mechanism that is dependent on an environment enriched by CD8 T cells (56). The authors demonstrated that CD8 T cells play an important role in protection after re-infection, producing high amounts of IFN-γ (56). CD8 T cells from healed mice were able to transfer DTH responses to naïve recipients (57) and also displayed cytotoxic activity (58). Contradicting these findings, it was demonstrated that CD8 T cells were not essential to the primary protective response to *L. major*-infected mice because β2-microgobulin-deficient or *Cd8*-/- mice maintained their capacity to resolve the primary infection (59, 60). In 2002, using an intradermal model of infection with low number of parasites (~100), Belkaid et al. demonstrated that CD8 T cells are important for the control of primary infections in resistant mice infected by *L. major* (61). In this low-infection model, CD8 T cells producing IFN-γ promoted the change from the early Th2 response toward a Th1 response (62).

CD8 T cells producing IFN-γ are also important for the modulation of the CD4 T cell response. Although the depletion of CD8 T cells did not interfere with the proliferative ability of CD4 T cells, a reduction in the percentage of CD4 T cells producing IFN-γ was observed, an effect that was associated with an increase in parasite load in mice, suggesting an interaction between CD4 and CD8 T cells (63). In human leishmaniasis, important roles of CD8 T cells in the healing process through IFN-γ production (25, 27, 64) and in resistance to the infection have been described (31). However, few reports have evaluated the role of these cells in the primary infection. Our group used an experimental approach called *in vitro* priming, in which cells from healthy volunteers were stimulated by *L. amazonensis* and cultured for 96 h. We observed that CD8 T cells were the first to express activation markers and were important for Th1 activation (65). In infection by *L. braziliensis*, an increase in the number of CD8 T cells reactive to *Leishmania* antigens during the healing process was observed (66); large proportion of these cells could also be observed at the inflammatory site of the infection (67). A higher amount of CD8 T cells producing IFN-γ was also associated with protective immunity in infection by *L. major* (68). However, the presence of CD8 T cells showing functional exhaustion has been observed in DCL patients: these cells produce a low level of IFN-γ upon stimulation compared to CL patients (69). Therefore, these cells would be important in the development of acquired immunity to the infection.

CD8 T cells are also able to produce IL-10. Bourreau et al. analyzed PBMCs from unexposed naïve subjects and CL patients in response to *Leishmania guyanensis* stimulation and demonstrated that IL-10 was produced by the cells from both groups when TGF-β was neutralized. An analysis of the phenotype of IL-10-producing cells in naïve subjects clearly showed that they are memory cells characterized as CD45RA^-^CD8 T cells (70) and appear to be effective during human infection.

Despite the apparent protective role of CD8 T cells following infection with the intracellular *Leishmania* parasite, these cells have been paradoxically linked to immunopathological responses. Our group implicated CD8 T cells in the pathogenesis of ML (71, 72). The presence of cytolytic CD8 T cells has also been demonstrated in the lesions from CL patients. Machado et al. observed the presence of CD8 T cells and a strong expression of a molecule associated with cytotoxic activity (TIA-1) in the inflammatory site of infection (73). Moreover, Faria et al. showed the recruitment of CD8 T cells expressing granzyme A to lesions of CL patients, and the expression of this protease was positively correlated with lesion progression in these patients (74). Our group found similar results, showing the recruitment of CD8 T cells to lesion sites in CL patients. These cells expressed granzyme B and CD107a and were positively correlated with the necrosis intensity and lesion size observed in these patients (38). More recently, Novais et al. showed that disease progression and metastasis in *L. braziliensis*-infected mice were directly associated with the presence of CD8 T cells. The authors demonstrated that perforin-expressing CD8 T cells were required to mediate immunopathology and that this was independent of the parasite burden (75). In human CL caused by *L. major* and *Leishmania mexicana*, granzyme B activity was also associated with a good prognosis (76, 77). In these studies, the *in vitro* cytotoxicity observed in the co-culture of *Leishmania* infected macrophages with peripheral blood lymphocytes appeared to be mediated by granzyme B. However, the release of granzyme B does not appear to participate in the control of parasite growth (38, 75). Recently, Crosby et al. demonstrated the participation of bystander memory CD8 T cells expressing NKG2D in leishmanial lesion progression. The authors showed that mice infected previously by viral or bacterial pathogens and challenged by *L. major* developed significantly larger lesions, with an increased number of NKG2D-positive CD8 T cells; however, the immunopathology was not associated with any changes in the parasite burden (78). In fact, non-*Leishmania*-specific CD8 T cells are found within human leishmanial lesions (79). Taken together, these studies highlight the harmful role played by cytolytic CD8 T cells in contributing to tissue injury. These data are summarized in Table 1.

**Table 1:** **Protective vs pathological function of CD8 T cells**.

**PROTECTIVE ROLE OF CD8 T CELLS**	
Depletion of CD4 T cells in mice makes them resistant to infection, a mechanism dependent on CD8 T cells	(56)
Transfer of CD8 T cells from healed mice is able to induce the DTH response in naïve recipients	(57)
Infection with a low number of parasites elicits a primary immune response by CD8 T cells	(61)
IFN-γ produced by CD8 T cells promotes the change from a Th2 to a Th1 response	(62)
CD8 T cells are important in the healing process and in resistance to the infection in human beings	(25, 27, 31, 64, 66)
*In vitro* priming experiments with human cells show that CD8 T cells produce IFN-γ and drive Th1 differentiation	(65)
CD8 T cells in infection by *L. mexicana* and *L. major* are related to a good prognosis in human beings	(77)
CD8 T cells produce IFN-γ in *L. major* infection	(68)
**PATHOLOGICAL ROLE OF CD8 T CELLS**	
CD8 T cells are not essential for the primary response to *L. major* in mice	(59, 60)
Cytotoxic cells are associated with pathogenesis in mucosal leishmaniasis	(71, 72)
CD8 T cells displaying cytotoxic activity are found at the inflammatory site of infection in human beings	(73)
CD8 T cells express granzyme A in the lesions of CL patients	(74)
CD8 T cells expressing granzyme B and CD107a, characteristic of CTL, are related to tissue damage in the lesions of CL patients	(38)
Disease progression and metastasis in *L. braziliensis*-infected mice are related to cytotoxic CD8 T cells.	(75)

## Concluding Remarks

Studies in the literature have shown the important role played by CD4 T cells in protection against human leishmaniasis by producing cytokines able to activate the macrophages that kill the parasites. However, a strong inflammatory response contributes to lesion development and the immunopathogenesis of the disease. More recently, the role displayed by CD8 T cells has been better characterized, mainly with regard to the infection caused by *L. braziliensis* in human beings and mice. These cells contribute to the differentiation of Th1 responses in the early events of parasite infection, whereas they contribute to lesion development after establishment of the infection. Their presence and cytotoxic activity are directly correlated to the lesion size and the presence of necrosis. Interestingly, after cure of the disease, CD8 T cells can produce IFN-γ and are also correlated with the healing process.

The understanding of the immunopathological mechanisms displayed by CD4 and CD8 T cells is essential for the design of new therapeutic and vaccine strategies in human TL.

## Conflict of Interest Statement

The authors declare that the research was conducted in the absence of any commercial or financial relationships that could be construed as a potential conflict of interest.
